# Variation in life history traits and transcriptome associated with adaptation to diet shifts in the ladybird *Cryptolaemus montrouzieri*

**DOI:** 10.1186/s12864-016-2611-8

**Published:** 2016-04-11

**Authors:** Hao-Sen Li, Chang Pan, Patrick De Clercq, Adam Ślipiński, Hong Pang

**Affiliations:** State Key Laboratory of Biocontrol, Ecology and Evolution, School of Life Sciences, Sun Yat-sen University, Guangzhou, 510275 Guangdong China; Department of Crop Protection, Faculty of Bioscience Engineering, Ghent University, Ghent, Belgium; Australian National Insect Collection, National Research Collections, CSIRO, Australia, GPO Box 1700, Canberra, ACT 2601 Australia

**Keywords:** Predacious ladybird, *Cryptolaemus montrouzieri*, Coccid, Aphid, Diet shift, Life history, Transcriptome

## Abstract

**Background:**

Despite the broad diet range of many predatory ladybirds, the mechanisms involved in their adaptation to diet shifts are not completely understood. Here, we explored how a primarily coccidophagous ladybird *Cryptolaemus montrouzieri* adapts to feeding on aphids.

**Results:**

Based on the lower survival rate, longer developmental time, and lower adult body weight and reproduction rate of the predator, the aphid *Megoura japonica* proved being less suitable to support *C. montrouzieri* as compared with the citrus mealybug *Planococcus citri*. The results indicated up-regulation of genes related to ribosome and translation in fourth instars, which may be related to their suboptimal development. Also, several genes related to biochemical transport and metabolism, and detoxification were up-regulated as a result of adaptation to the changes in nutritional and non-nutritional (toxic) components of the prey.

**Conclusion:**

Our results indicated that *C. montrouzieri* succeeded in feeding on aphids by regulation of genes related to development, digestion and detoxification. Thus, we argue that these candidate genes are valuable for further studies of the functional evolution of ladybirds led by diet shifts.

**Electronic supplementary material:**

The online version of this article (doi:10.1186/s12864-016-2611-8) contains supplementary material, which is available to authorized users.

## Background

The predatory ladybirds (Coleoptera, Coccinellidae) include many beneficial and economically significant species that are used as biological control agents against insect pests. Besides various insects and mites, many ladybirds can also feed on plant materials and fungi [1, 2]. Coccids (scale insects and mealybugs) and aphids are two of the most important prey types of predatory ladybirds. Globally, coccids are the dominant prey groups of 36 % of Coccinellidae species, whereas 20 % prey primarily on aphids [3]. Evolutionary studies of the family Coccinellidae have suggested that ancestral ladybirds have switched from fungi to coccids [1]. Several clades have subsequently adapted to feeding on aphids or have then switched back to feeding on coccids [1]. Coccids and aphids have a quite different biochemical composition and possess different secondary metabolic products for self-protection [4], although they are closely related in systematics. Thus, the mechanisms of adaptation to these two diets and of diet shifts in ladybirds should be unique, but they are still incompletely understood [1]. As a result, the evolutionary processes of ladybirds led by diet shifts remain unclear.

Identifying transcriptional changes associated with the early stages of diet shifts is an important step in understanding the role of transcriptional plasticity and subsequent gene evolution in facilitating adaptation [5, 6]. Previous studies have demonstrated transcriptional regulations associated with insects feeding on different plant diets, and these regulations were commonly detected in ribosomal proteins, detoxification enzymes and digestive proteases [5, 7, 8]. However, relatively few studies have examined such mechanisms in ladybirds or other insect predators [9].

*Cryptolaemus montrouzieri* (also known as the mealybug destroyer) is native to Australia but is used worldwide as biological control agent. This species provides an opportunity to study the above issue because it mainly feeds on coccid species in the field, while it can also feed on aphids, whiteflies and eggs of moths or other ladybirds under laboratory conditions [10]. These non-coccid diets are overall less suitable for survival, development and reproduction of *C. montrouzieri*, but often suffice for completing its life cycle [10]. In the present study, we explored the mechanisms involved in diet change from coccids to aphids by this coccidophagous ladybird. A laboratory population of *C. montrouzieri* that was maintained on the citrus mealybug *Planococcus citri* for several years was artificially transferred to the aphid *Megoura japonica*, a common aphid pest in China [11]. We first compared the life history traits of *C. montrouzieri* feeding on these two foods, which allowed us to test their suitability to support development and reproduction. To investigate how transcriptional variation may contribute to adaptation to a new food, we then compared gene expression among the two diet treatments in order to find differentially expressed genes (DEGs) in response to the diet shift.

## Results

### Comparison of life history traits

Developmental and reproductive parameters of *C. montrouzieri* feeding on *M. japonica* versus *P. citri* are compared in Table 1. *C. montrouzieri* feeding on aphids had a significantly longer development time in each larval instar and in the pupal stage, as compared with those offered mealybug prey (U_(18,27)_ = 0.0, *p* < 0.001; U_(18,24)_ = 112.0, *p* = 0.002; U_(17,27)_ =51.0, *p* < 0.001; U_(18,27)_ = 22.5, *p* < 0.001; U_(18,27)_ = 54.0, *p* < 0.001; U_(18,27)_ = 18.0, *p* < 0.001 from first instar to pupa respectively by Mann–Whitney U test). Also, the aphid-fed larvae had a significantly lower survival rate in the first, third and fourth instar (χ^2^ = 41.270, df = 1, *p* < 0.001 for first instar, χ^2^ = 8.606, df = 1, *p* = 0.003 for third instar and χ^2^ = 6.363, df = 1, *p* = 0.012 for fourth instar by logistic regression). Particularly in the first instar, survival on aphids was poor (33 %). The body weights of both adult females and males were significantly lower on aphid prey than on mealybugs (*p* < 0.001 by t-test), as was the proportion of adult females emerging (χ^2^ = 10.078, df = 1, *p* = 0.002 by logistic regression). Female adults maintained on aphids had significantly longer periods of preoviposition (*p* < 0.001 by t-test) and oviposition (*p* = 0.020 by t-test). The number of deposited eggs was significantly lower on aphids (*p* < 0.001 by t-test) and was nearly half of that on mealybugs. Eggs deposited by aphid-fed females had a lower hatching rate than those deposited by mealybug-fed females, but this difference was not significant.

**Table 1:** Life history traits (means ± SE) of *C. montrouzieri* feeding on aphids (*M. japonica*) and mealybugs (*P. citri*)

Life history traits	Diet treatments	
	*M. japonica*	*P. citri*
No. of tested individuals	56	48
Development time of first instar (days)^a^	6.61 ± 0.128	3.78 ± 0.08
Development time of second instar (days)^a^	4.00 ± 0.11	3.46 ± 0.10
Development time of third instar (days)^a^	4.76 ± 0.16	3.43 ± 0.10
Development time of fourth instar (days)^a^	6.78 ± 0.18	5.42 ± 0.09
Development time of prepupa (days)^a^	6.27 ± 0.20	5.03 ± 0.10
Development time of pupa (days)^a^	8.67 ± 0.24	6.31 ± 0.13
Survival rate of first instar (%)^a^	33.33	90.00
Survival rate of second instar (%)	76.00	88.89
Survival rate of third instar (%)^a^	83.33	94.23
Survival rate of fourth instar (%)^a^	85.71	94.12
Survival rate of pupa (%)	96.30	87.50
Weight of female adult (mg)^a^	7.59 ± 0.16	10.94 ± 0.29
Weight of male adult (mg)^a^	6.72 ± 0.24	9.17 ± 0.42
Female ratio (%)^a^	58.33	67.22
Preoviposition time (days)^a^	22.83 ± 2.12	5.29 ± 0.18
Oviposition time (days)^a^	88.60 ± 3.56	76.57 ± 2.69
Postoviposition time (days)	11.20 ± 0.91	10.86 ± 0.55
No. of deposited eggs^a^	196.57 ± 9.75	366.57 ± 12.43
Egg hatch rate (%)	67.12	75.00

Traits with an asterisk are significantly different (*p* < 0.050, binary logical regression, Student’s t-test or Mann–Whitney U test)

### Sequence data processing

We sequenced eight transcriptome libraries, which were from *C. montrouzieri* fourth instar larvae feeding on mealybugs (LM1 and LM2), fourth instar larvae feeding on aphids (LA1 and LA2), female adults feeding on mealybugs (AM1 and AM2) and female adults feeding on aphids (AA1 and AA2). Each of them had 20–30 million high-quality reads comprised of 5–7 billion nucleotides (5–7 GB) (Additional file 1: Table S1). These reads were assembled into 20,006,931 contigs, 122,193 transcripts and 73,655 unigenes, with 31.57 % of the unigenes being more than 500 bp and 17.42 % of the unigenes more than 1000 bp (Additional file 2: Figure S1, Additional file 1: Table S2). Of these unigenes, 28,559 (38.77 %) were annotated in the National Center for Biotechnology Information (NCBI) non-redundant dataset (nr), Swissprot, Cluster of Orthologous Groups (COG), EuKaryotic Orthologous Groups (KOG), Pfam, Gene Ontology (GO) or Kyoto Encyclopedia of Genes and Genomes (KEGG) (Additional file 1: Table S3). The highest percentage of *C. montrouzieri* sequences were matched to *Tribolium castaneum* (40 %). The FPKM (fragments per kilobase of transcript per million mapped reads) density had a similar pattern within each sample of two individuals (stage/diet) (Additional file 3: Figure S2), indicating that our transcriptome analysis of each treatment was highly reproducible.

### Transcriptional responses to diet shift

An overall view of gene expressions in the eight transcriptome libraries of *C. montrouzieri* is presented in the hierarchical clustering heat map in Additional file 4: Figure S3 and in the summary of DEGs in Additional file 1: Table S4, with details of the fourth instar in Additional file 5: Table S5 and adult stage in Additional file 6: Table S6, respectively. As shown, their gene expressions were predominantly affected by development stage, followed by diet. In this study, we mainly focused on the effect of diet shifts. So, the following DEG analysis would only be performed in LM vs LA and AM vs AA, respectively. In fourth instar larvae, a total of 788 DEGs were observed in the aphid-feeding lines compared with the mealybug-feeding lines, among which 449 were up-regulated and 339 were down-regulated, while fewer DEGs were observed in the adult stage. For the diet treatments of adults, the total number of DEGs was 331, with 224 being up-regulated and 87 down-regulated. In addition, 68 genes were differentially expressed in both the larval and adult stage (Additional file 1: Table S4).

**Fig. 1:**
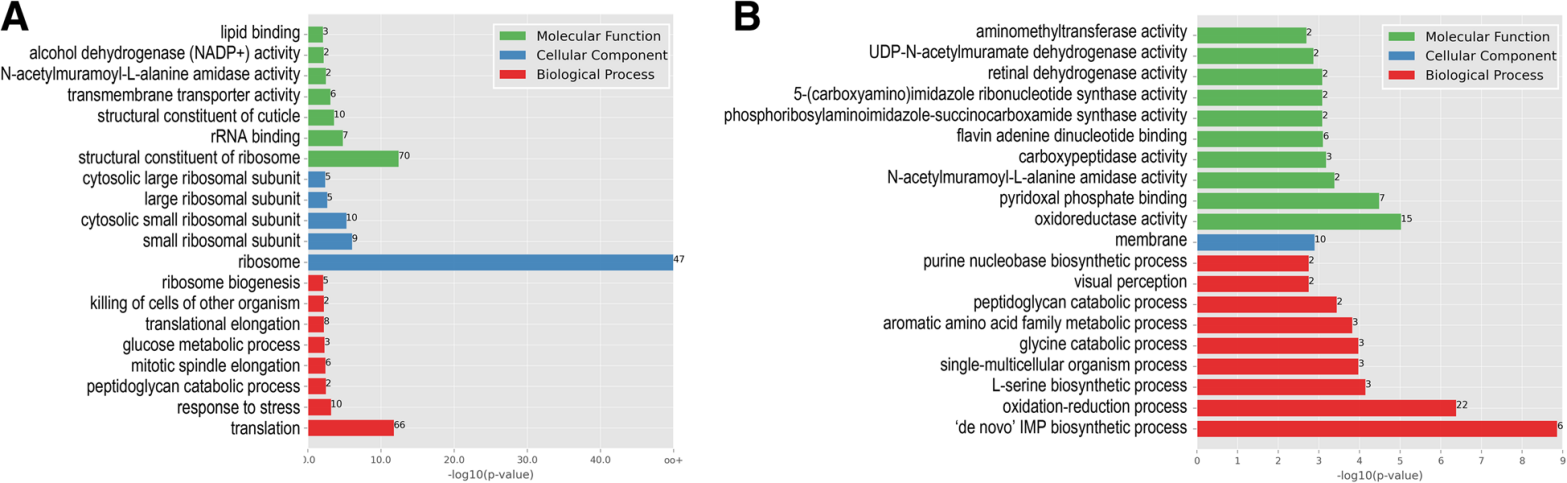
Gene ontology (GO) enrichment of differentially expressed genes (DEGs) in **a** fourth instar larva and **b** adult female. The 20 most enriched GO terms are shown together with their -log10(*p*-value) and number of genes (right of bars)

In fourth instar larvae, the strongest changes in the aphid-feeding lines were in the ribosome of cellular component (GO) categories, followed by structural constituent of ribosome of molecular function GO categories and translation of biological process GO categories (Fig. 1a). These three changes were considered significant, with –log10(*p*-value) higher than 10. Among all of the DEGs in fourth instar larvae, 79 were coding the large 60S and small 40S ribosomal proteins, with 52 being up-regulated and 27 down-regulated (Table 2 and Additional file 7: Table S7 in detail). In the adult stage, no DEGs related to ribosome were detected. In this stage, the strongest changes were in the ‘de novo’ integral membrane protein (IMP) biosynthetic process and oxidation-reduction process of biological process GO categories, followed by oxidoreductase activity of molecular function GO categories (Fig. 1b). Only one cellular component GO category was in the top 20 of the strongest changes. However, these changes in the adult stage were not significant, with –log10(*p*-value) lower than 10.

**Table 2:** Summary of candidate differentially expressed genes (DEGs) related to ribosome, biochemical transport and metabolism and detoxification

	Candidate genes	Larva			Adult		
		DEG	Up	Down	DEG	Up	Down
Ribosome	Ribosomal protein	79	52	27	0	0	0
Biochemical transport and metabolism	Carbohydrate	34	25	9	15	15	0
	Lipid	31	7	24	25	14	11
	Amino acid	22	17	5	21	21	0
	Inorganic ion	12	7	5	4	3	1
	Nucleotide	4	2	2	10	10	0
	Coenzyme	4	2	2	4	3	1
Detoxification	P450	16	13	3	8	8	0
	GST	2	1	1	1	1	0
	UGT	3	2	1	3	3	0
	CE	3	2	1	3	3	0

*P450*: cytochrome P450 monooxygenases, *GST*: glutathione S-transferases, *UGT*: UDP-glycosyltransferases, *CE*: carboxylesterases

**Fig. 2:**
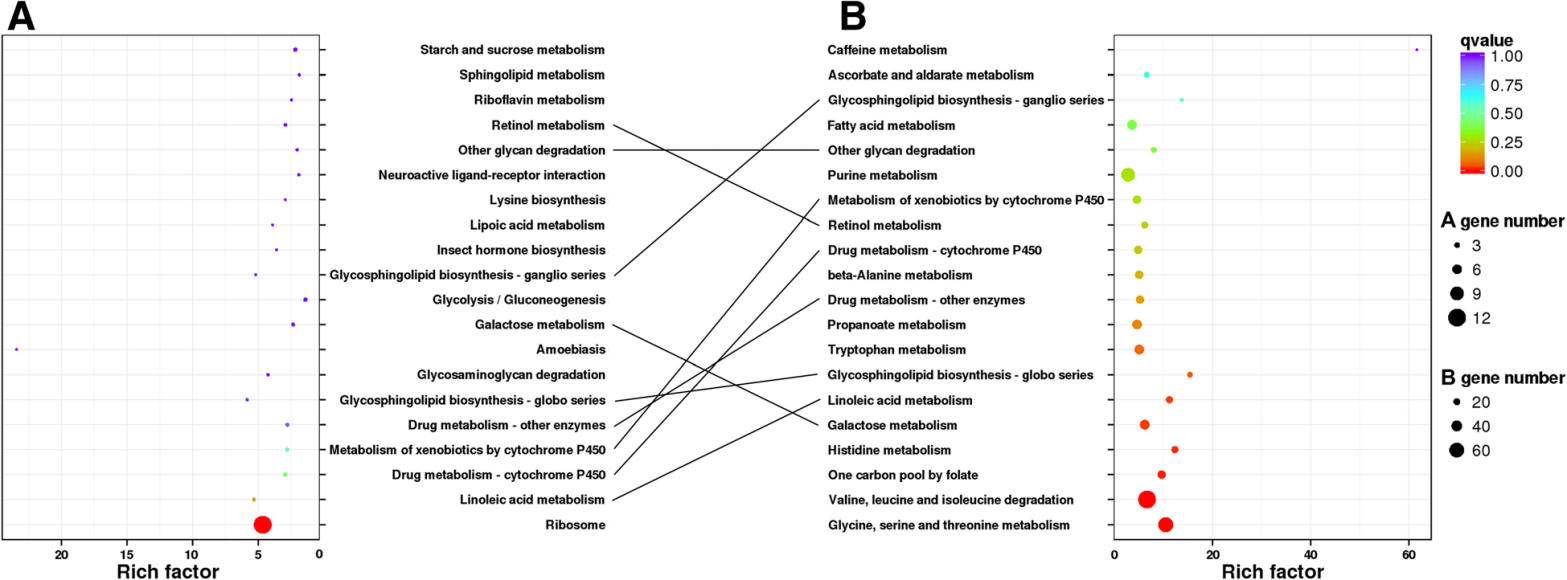
Kyoto Encyclopedia of Genes and Genomes (KEGG) pathways enrichment of differentially expressed genes (DEGs) in **a** fourth instar larva and **b** adult female. The 20 most enriched KEGG pathways are shown together with their q-value (color), rich factor (vertical ordinate) and number of genes (size of circles)

In fourth instar larvae, the genes that were differentially expressed in the aphid-feeding lines were involved in 50 KEGG pathways, mainly involving ribosome, linoleic acid metabolism and drug metabolism-cytochrome P450 (Fig. 2a). In the adult stage, the genes that were differentially expressed in the aphid-feeding lines were involved in 63 KEGG pathways, mainly involving glycine, serine and threonine metabolism, valine, leucine and isoleucine degradation and one carbon pool folate (Fig. 2b). Among the top 20 of the strongest changed pathways, eight were common in fourth instar larvae and adults, with four being related to biochemical (lipid and carbohydrate) metabolism and three being related to detoxification. According to the results of KOG annotations of transport and metabolism of six key biochemicals, most of the DEGs related to carbohydrate and amino acid transport and metabolism were up-regulated, while DEGs related to lipid transport and metabolism were both up-regulated and down-regulated (Table 2, and Additional file 7: Table S7, Additional file 8: Table S8 in detail). Among the detoxifying genes, cytochrome P450 monooxygenases (P450s), glutathione S-transferases (GSTs), UDP-glycosyltransferases (UGTs) and carboxylesterases (CEs) were commonly reported in previous studies [6–8]. In this study, most of the detoxification-related DEGs were P450s. All of these detoxification-related DEGs were mainly up-regulated (Table 2, and Additional file 7: Table S7, Additional file 8: Table S8 in detail).

Twenty-one DEGs of fourth instars and 19 of female adults were randomly selected to validate the expression profiles obtained with the transcriptome analysis. All of them yielded quantitative Real-Time PCR (qRT-PCR) products, whose sequences perfectly matched the up or down-regulations of transcriptome analysis (Additional file 9: Figure S4). These results demonstrate the reliability of the results of the transcriptome analysis.

## Discussion

Diet is a major determinant of physiological performance in insects [5], and diet shifts can involve changes associated with life history, physiological and behavioral traits [5, 12–14]. With the development of next generation sequencing methods, studies have begun to identify genes under regulation or evolution associated with diet shifts in insects [7, 9, 15–18]. Furthermore, the relationship between changes in life history traits and transcriptome can help us to better understand the mechanism of adaptation to diet shifts [19]. Also, although little attention has been directed towards analyzing transcriptional adaptation of insect predators to diet shifts, recent comparative gene expression studies of phytophagous insects or mites adapted to different host plants [5, 7, 18, 20, 21] and predatory insects adapted to an artificial diet [17] provide a basis for comparison to the findings of our study.

### Changes in life history traits

In this study, *C. montrouzieri* feeding on either mealybugs or aphids successfully completed their life cycles. Although *C. montrouzieri* has long been considered as a mealybug specialist [22], a current host range test indicated that *C. montrouzieri* can also be maintained on several non-coccid prey species under laboratory conditions [10]. However, such diet shifts significantly changed some of the life history traits in both the larval and adult stages of *C. montrouzieri* [9]. In this study, all of the changes in development, survival and reproduction were adverse, suggesting that a diet of aphids would be less suitable for this coccidophagous ladybird than mealybugs. This unsuitability of aphids to *C. montrouzieri* can possibly be attributed to differences in the biochemical composition, body size and mobility of the prey and tritrophic effects caused by the different host plant-prey associations [10]. Also, these changes in life history traits possibly reflected differential expression of related genes in adaptation to diet shift.

### DEGs related to ribosome and translation

In this study, the most remarkable change in gene expression of *C. montrouzieri* feeding on aphids is in ribosome and translation. The results of DEG enrichments suggested that genes related to ribosome and translation, mainly coding ribosomal proteins of 40S and 60S, were specifically over-represented after the diet shift in the fourth instar larvae, and most of these genes were up-regulated. Ribosome synthesis and translation are usually at high rates during development [23, 24]. As such change was not detected in the adult stage, we note that the factors of DEGs related to ribosome and translation are important for the development of larvae. On the other hand, ribosomal proteins are mostly associated with their standard role in protein translation and genes encoding them have been considered as stably expressed ‘housekeeping’ genes. Nevertheless, these genes also have been reported to be differentially regulated in *Polygonia c-album* [8], *Heliothis virescens* [25], *Bemisia tabaci* [26] and *Helicoverpa armigera* [27] when feeding on unsuitable diets, and mostly they were also up-regulated. In an insect-plant system, these differential regulations in insects are considered to counteract the ribosome-inactivating proteins, which are insecticidal proteins in certain plants [8, 28]. Although no ribosome-inactivating protein has been reported in aphids or mealybugs, it is possible that prey contain similar proteins, which activate the regulation of genes related to ribosome and translation in their predators. However, there are no previous reports on such regulation in predator–prey systems, and such regulation and its mechanism are worthy to be investigated in the future.

### DEGs related to biochemical transport and metabolism

Although coccids and aphids are from the same order (Hemiptera), they are expected to differ in biochemical composition [4] and thus have a different nutritional value for ladybird predators. There is evidence for some differentially expressed metabolism-related genes of insects in adapting to different biochemical composition of diets [9, 17, 29]. In this study, the different composition of the two diets was also reflected in the transcriptional changes of biochemical metabolism we observed in *C. montrouzieri*. Among the transport and metabolism of six key biochemicals, the three energy related chemical groups (carbohydrate, lipid and amino acid) had the highest number of DEGs, and the DEGs related to carbohydrate and amino acid were mainly up-regulated in both life stages. These results suggest that key nutrients for *C. montrouzieri* might be lacking when feeding on aphids, so that their transport and metabolism need to be accordingly controlled by gene regulation. Although there are some data on the nutritional biology of the more plastic aphidophagous ladybirds [30], there is little or no information on the nutrient requirements of the more specialized coccid-feeding ladybirds. An alternative explanation may lie in tritrophic effects related to the different food of the aphids and mealybugs. Our study only provides a general view of the transcriptional changes in biochemical transport and metabolism in response to diet shift, and an organ/tissue specific experiment (e.g. the midgut which is the major organ for diet degradation and nutrient absorption) could help to detect a more accurate response.

### DEGs related to detoxification

Besides their nutritional requirements, insects should at the same time be able to cope with toxic chemicals from their diets. When confronted with diet shifts, insects can successfully survive on their new diet through enzymes coded by detoxifying genes, among which P450s, GSTs, UGTs and CEs have been widely reported [6–8]. In this study, the genes in several detoxification pathways were significantly regulated in both fourth instar larvae and adults of *C. montrouzieri*. Among these pathways, we detected that several detoxification-related genes, especially the P450s, were differentially regulated and mainly up-regulated in both life stages when feeding on aphids as compared with mealybugs. Eleven P450s, one UGT and two CEs were up-regulated and one UGT was down-regulated commonly in both fourth instar larvae and adults, suggesting that these genes might be more important in detoxification during diet shifts than the others. The expression of P450 genes can control the transformation of a number of xenobiotics in herbivorous insects [31, 32], and is reported to be involved in the detoxification of pesticides in *C. montrouzieri* [33] and another ladybird *Propylea japonica* [34]. These results, together with lower survival rates of the larval stage, suggest that *C. montrouzieri* might succeed in feeding on aphids by eliminating or transforming toxic chemicals from the prey, and the up-regulation of P450 or other detoxification-related genes could play an important role in this process.

### Possibility of diet-associated evolution

Diet shift and subsequent adaptation can drive evolution and diversity of fast-evolved arthropods [6, 35, 36]. In this study, despite the fact that a large number of genes were regulated in adaptation to a new diet, the individuals of *C. montrouzieri* feeding on aphids had a lower developmental and reproductive performance than those given mealybugs. Directional selection is expected to further alter the plastic response in the direction of the optimum, and thus results in adaptive evolution (known as the Baldwin effect, [6, 37–39]). In this case, if ladybirds sustain in a long-term feeding on a new diet, the DEGs which were initially beneficial in this diet shift may subsequently evolve. For instance, the detoxification gene *GstD1* of *Drosophila melanogaster* was under positive selection among populations feeding on different diets [40], and this gene was first differentially regulated in early diet shifts [41]. Thus, the DEGs detected in this study could be further used to explore the diet-associated evolution within *C. montrouzieri* and other Coccinellidae.

## Conclusions

In this study, we explored how a ladybird adapted to diet shift using comparisons of life history traits and transcriptome profiles. We showed that the new diets were less suitable to the ladybirds. The up-regulation of genes related to ribosome and translation in instars may be related to their suboptimal development. In addition, the up-regulation of genes related to biochemical transport and metabolism, and detoxification were probably as a result of adaptation to the changes in nutritional and non-nutritional (toxic) components of the prey. In the future, these candidate genes are valuable for the studies of the functional evolution of ladybirds led by diet shifts.

## Methods

### Laboratory rearing of ladybirds and their prey

Individuals of the ladybird and its prey used in the present study were obtained from a laboratory-reared population at Sun Yat-sen University, Guangzhou, China which was maintained in the laboratory for more than ten years before the onset of this project. *C. montrouzieri* was maintained in RXZ-310B containers (Jiangnan Instrument, Ningbo, China) on *P. citri* mealybugs which were produced on fruits of pumpkin, *Cucurbita moschata. M. japonica* was maintained on plants of horsebean, *Vicia faba* in cages. All insects and plants were kept in climate chambers 27 °C, a 80 % relative humidity (RH), and a 14:10 (L:D) h photoperiod.

### Comparison of life history traits

The life history of *C. montrouzieri* feeding on *P. citri* or *M. japonica* was investigated by offering the ladybird the same prey species during its larval and adult stages. Approximately 60 first instars of *C. montrouzieri* (<24 h old) for each diet treatment were placed individually in plastic Petri dishes (diameter 5 cm, height 2 cm). Both prey types were offered ad libitum and replenished daily. Survival and development of *C. montrouzieri* larvae were monitored daily. Newly emerged adults were sexed and weighed, and then males and females were paired. The oviposition substrate (a piece of cotton (~1 × 1 cm)) was checked daily for eggs to determine the preoviposition period. Once the first egg was laid, substrates were replaced every three days throughout the total oviposition period. Egg hatch rate was monitored for 76 and 45 eggs in the aphid and mealybug diet treatments, respectively. All experiments were performed in a climatic chamber set at 27 °C and 80 % RH, and a 14:10 (L:D) h photoperiod.

Survival rates, egg hatch rates and sex ratio of the predators were compared through a binary logistic regression. A Kolmogorov-Smirnov test indicated that male and female body weight, oviposition times and number of deposited eggs of the predator were normally distributed and therefore analyzed using a Student’s T-test. As a Levene test indicated homoscedasticity, the means were separated using Tukey tests. According to a Kolmogorov-Smirnov test, larval developmental times were not normally distributed. Therefore, we used the non-parametric Mann–Whitney U test to evaluate differences in developmental time among treatments. In all tests, *P*-values below 0.05 were considered significant. All data were analyzed using SPSS 17.0 (SPSS Inc.).

### Comparison of transcriptomes

Fourth instar larvae (<24 h old) and female adults (~30 days old and fertile) of *C. montrouzieri* feeding on *P. citri* or *M. japonica* were collected for the following transcriptome comparison. For each life stage and diet treatment, two individuals were randomly collected from the above life history experiment. After ~12 h of starvation, the total RNA of these eight individuals was extracted using TRIzon reagent (CWBIO, Beijing, China) according to the protocol of the manufacturer. RNA quality and quantity was determined using a Nanodrop 1000 spectrophotometer (Thermo Fisher Scientific, Wilmington, DE) and Bioanalyzer RNA nano chip (Agilent Technologies, Singapore). Only the RNA samples with 260/280 ratio from 1.8 to 2.0, 260/230 ratio from 2.0 to 2.5 and RIN (RNA integrity number) of more than 8.0, were used for sequencing.

Approximately 20 μg of total RNA for one individual was used for the construction of libraries using the mRNA-Seq Sample Prep kit (Illumina Inc., San Diego, CA) according to the protocol of the manufacturer. Equal quantities of libraries (approximately 5 ng per sample) with different indices were mixed and stored in a freezer at −80 °C before sequencing. Sequencing was performed in a v3 flowcell on an Illumina HiSeq 2500 sequencer, using the TruSeq Paired-End Cluster Kit v3 (Illumina PE-401–3001) and the TruSeq SBS HS Kit v3 at 200 cycles (Illumina FC-401–3001), generating 2 × 125 bp reads. Image analysis and base calling was done using the HiSeq Control Software version 1.4 and the Off-Line Base Caller v1.9. About 120 million high quality RNA-Seq reads (with a quality score of >20 for each base) were pooled from Illumina sequencing of each of the eight samples and were then assembled into contigs using Trinity [42]. All sequencing data were deposited in the NCBI Short Read Archive (SRA) under BioProject ID PRJNA304936 (BioSample accession number: LM1: SAMN04311931; LM2: SAMN04311932; LA1: SAMN04309303; LA2: SAMN04311923; AM1: SAMN04311933; AM2: SAMN04311935; AA1: SAMN04311934; AA2: SAMN04311936). We quantified transcript levels in RPKM (reads per kilobase of exon mode per million mapped reads) [43]. K-Means clustering was performed by the Euclidean distance method and each centroid was the mean of the points in that cluster. Hierarchical clustering of gene expression was performed by the clustergram function in the Matlab Bioinformatics toolbox with default settings. The FPKM method was used to calculate unigene expression [44].

The unigenes were searched against nr, Swissprot, COG, KOG and Pfam in BlastX with a cut-off E-value of 10e^−5^. The results of BlastX annotation were uploaded on Blast2GO to generate GO annotations and mapped to the categories of GO database, and also searched against the KEGG pathway [45]. To investigate which GO terms and KEGG pathways the DEGs participated in, all of the clustered DEGs were mapped back to the GO and KEGG databases. The statistical significances of the GO enrichment were evaluated by the hypergeometric distribution testing:

$$ \mathrm{p}=1\ \hbox{-}\;{\displaystyle \sum_{i=0}^{m-1}\frac{\left(\begin{array}{l}M\\ {}i\end{array}\right)\left(\begin{array}{l}N-M\\ {}n-i\end{array}\right)}{\left(\begin{array}{l}N\\ {}n\end{array}\right)}} $$

Where N is the number of unigenes with GO annotation, n is the number of DEGs with GO annotation, M is the number of unigenes with one specific GO annotation and m is the number of differently expressed unigenes with one specific GO annotation [46]. In the case of statistical significance of the KEGG enrichment, the rich factors were calculated by (DEG number/number of genes annotated by KEGG)/(number of DEG in pathway/number of genes in pathway).

To confirm the results of transcriptome comparisons, the qRT-PCRs were performed on randomly selected genes which were differentially expressed (21 DEGs for the four instar larvae and 19 DEGs for the adults). The concentration of each RNA sample was adjusted to 1 mg/ml with nuclease-free water, and ca. 6 μg of total RNA was used as the template to synthesize first-strand cDNA in a 20 μl reaction system using a Superscript III Reverse Transcriptase kit (Invitrogen) following the protocol of manufacturer. The sequences of the specific primer sets are listed in Additional file 1: Table S9. The *β-Actin* gene of *C. montrouzieri* was used as an internal gene. The quantitative real-time PCRs were performed using the SYBR (R) Green I Nucleic A kit (Invitrogen) according to the protocol of the manufacturer. The cycling parameters were 95 °C for 2 min followed by 40 cycles at 95 °C for 10 s and 60 °C for 30 s ending with a melting curve analysis (60 °C to 95 °C in increments of 0.5 °C every 5 s) to check for nonspecific product amplification. Relative gene expression was analyzed by the 2^-ΔΔCt^ method [47].

### Ethics approval and consent to participate

Not applicable.

### Consent for publication

Not applicable.

### Availability of data and material

The data sets supporting the results of this article are included within the article and its additional files.

## Additional files

Additional file 1: Table S1. Summary of the sequence reads obtained from eight transcriptome libraries of *C. montrouzieri*. Eight transcriptome libraries are from fourth instar larvae feeding on mealybugs (LM1 and LM2), fourth instar larvae feeding on aphids (LA1 and LA2), female adults feeding on mealybugs (AM1 and AM2) and female adults feeding on aphids (AA1 and AA2). **Table S2.** Summary of the assembled contigs, transcripts and unigenes obtained from the combined reads of eight transcriptome libraries of *C. montrouzieri*. **Table S3.** Summary of integrated annotations of unigenes and differentially expressed genes (DEGs). **Table S4.** Summary of the differentially expressed genes (DEGs). **Table S9.** Quantitative Real-Time PCR (qRT-PCR) primers used in this study. (DOCX 20 kb) Additional file 2: Figure S1. Length distribution of the assembled A. contigs, B. transcripts and C. unigenes obtained from the combined reads of eight transcriptome libraries of *C. montrouzieri*. (TIF 774 kb) Additional file 3: Figure S2. Plot of fragments per kilobase of transcript per million mapped reads (FPKM) density of eight transcriptome libraries of *C. montrouzieri*. (TIF 804 kb) Additional file 4: Figure S3. Hierarchical clustering heat map of the gene abundance in eight transcriptome libraries of *C. montrouzieri*. Colours from red to blue represent the gene expression abundance from rich to poor. (TIF 1057 kb) Additional file 5: Table S5. Annotation of differentially expressed genes (DEGs) in comparison of fourth instar larvae feeding on mealybugs and aphids (LM vs LA). (XLS 353 kb) Additional file 6: Table S6. Annotation of differentially expressed genes (DEGs) in comparison of female adults feeding on mealybugs and aphids (AM vs AA). (XLS 149 kb) Additional file 7: Table S7. Candidate differentially expressed genes (DEGs) in comparison of fourth instar larvae feeding on mealybugs and aphids (LM vs LA). (XLS 126 kb) Additional file 8: Table S8. Candidate differentially expressed genes (DEGs) in comparison of female adults feeding on mealybugs and aphids (AM vs AA). (XLS 103 kb) Additional file 9: Figure S4. Verification of differentially expressed genes (DEG) by quantitative Real-Time PCR (qRT-PCR). DEG data in transcriptome analysis of fourth instar larva (A) and adult female (B) and qRT-PCR analysis of fourth instar larva (C) and adult female (D) are compared. Fold differences in the expression of selected genes in response to diet shifts were calculated using the 2^-ΔΔCt^ method. Data are presented as mean ± SD values of replicates for each gene transcript. Information of each gene number (1–21 of A and C and 1–19 of B and D) is shown in Additional file 1: Table S9. (TIF 656 kb)

## Abbreviations

CE: carboxylesterase
COG: cluster of orthologous groups
DEG: differentially expressed gene
FPKM: fragments per kilobase of transcript per million mapped reads
GO: gene ontology
GST: glutathione S-transferase
IMP: integral membrane protein
KEGG: Kyoto Encyclopedia of genes and Genomes
KOG: EuKaryotic orthologous groups
NCBI: national center for biotechnology information
nr: non-redundant dataset
P450: cytochrome P450 monooxygenase
qRT-PCR: quantitative Real-Time PCR
SRA: short read archive
UGT: UDP-glycosyltransferase
