# Supplementary material for: Variation in life history traits and transcriptome associated with adaptation to diet shifts in the ladybird Cryptolaemus montrouzieri
Source: BMC Genomics. 2016 Apr 11;17:281. doi: 10.1186/s12864-016-2611-8 (PMC4827204; doi:10.1186/s12864-016-2611-8)
Supplement: Additional file 1: Table S1. — Summary of the sequence reads obtained from eight transcriptome libraries of C. montrouzieri. Eight transcriptome libraries are from fourth instar larvae feeding on mealybugs (LM1 and LM2), fourth instar larvae feeding on aphids (LA1 and LA2), female adults feeding on mealybugs (AM1 and AM2) and female adults feeding on aphids (AA1 and AA2). Table S2. Summary of the assembled contigs, transcripts and unigenes obtained from the combined reads of eight transcriptome libraries of C. montrouzieri. Table S3. Summary of integrated annotations of unigenes and differentially expressed genes (DEGs). Table S4. Summary of the differentially expressed genes (DEGs). Table S9. Quantitative Real-Time PCR (qRT-PCR) primers used in this study. (DOCX 20 kb) [file 12864_2016_2611_MOESM1_ESM.docx]

Table S1. Summary of the sequence reads obtained from eight transcriptome libraries of *C. montrouzieri*. Eight transcriptome libraries are from fourth instar larvae feeding on mealybugs (LM1 and LM2), fourth instar larvae feeding on aphids (LA1 and LA2), female adults feeding on mealybugs (AM1 and AM2) and female adults feeding on aphids (AA1 and AA2).

| ID | reads | bases | GC (%) | N (%) | Q20 (%) | CycleQ20 (%) | Q30 (%) |
| --- | --- | --- | --- | --- | --- | --- | --- |
| LM1 | 21469120 | 5404317588 | 40.44 | 0.01 | 96.67 | 100.00 | 93.75 |
| LM2 | 22691614 | 5711365072 | 39.70 | 0.01 | 96.82 | 100.00 | 94.01 |
| LA1 | 21348934 | 5375471136 | 38.79 | 0.01 | 96.78 | 100.00 | 93.73 |
| LA2 | 21606620 | 5438740393 | 38.76 | 0.01 | 96.92 | 100.00 | 94.20 |
| AM1 | 21504336 | 5413556234 | 39.52 | 0.01 | 96.81 | 100.00 | 94.00 |
| AM2 | 30013056 | 7560540431 | 38.89 | 0.00 | 93.80 | 100.00 | 88.94 |
| AA1 | 23355761 | 5878508245 | 39.54 | 0.01 | 96.71 | 100.00 | 93.82 |
| AA2 | 29441976 | 7416882407 | 38.56 | 0.00 | 93.92 | 100.00 | 89.18 |

Table S2. Summary of the assembled contigs, transcripts and unigenes obtained from the combined reads of eight transcriptome libraries of *C. montrouzieri*.

| Length range | Contigs | Transcripts | Unigenes |
| --- | --- | --- | --- |
| 200-300 | 38892 (0.19%) | 41028 (33.58%) | 33537 (45.53%) |
| 300-500 | 19891 (0.10%) | 24542 (20.08%) | 16863 (22.89%) |
| 500-1000 | 12301 (0.06%) | 19930 (16.31%) | 10421 (14.15%) |
| 1000-2000 | 7238 (0.04%) | 16434 (13.45%) | 6636 (9.01%) |
| >2000 | 3037 (0.02%) | 20256 (16.58%) | 6196 (8.41%) |
| Total number | 20006931 | 122193 | 73655 |
| Total length | 783431804 | 137973558 | 54264509 |
| N50 length | 41 | 2544 | 1566 |
| Mean length | 39.16 | 1129.14 | 736.74 |

Table S3. Summary of integrated annotations of unigenes and differentially expressed genes (DEGs).

| Database | Number | 300<= <1000 | >=1000 | LM vs LA | AM vs AA | LM vs AM | LA vs AA |
| --- | --- | --- | --- | --- | --- | --- | --- |
| nr | 28044 | 9684 | 10433 | 681 | 277 | 3536 | 2042 |
| Swissprot | 15029 | 4441 | 7474 | 535 | 215 | 2736 | 1535 |
| COG | 7352 | 1996 | 3752 | 330 | 141 | 1392 | 791 |
| KOG | 15361 | 4318 | 7319 | 456 | 191 | 2483 | 1368 |
| Pfam | 15556 | 4532 | 8046 | 538 | 217 | 2844 | 1582 |
| GO | 11791 | 3635 | 4844 | 370 | 137 | 1672 | 919 |
| KEGG | 7319 | 2145 | 3236 | 245 | 92 | 1096 | 569 |
| All | 28559 | 9822 | 10458 | 687 | 282 | 3556 | 2053 |

Table S4. Summary of the differentially expressed genes (DEGs).

|  | Total | Up-regulated | Down-regulated |
| --- | --- | --- | --- |
| LM vs LA | 788 | 449 | 339 |
| AM vs AA | 331 | 244 | 87 |
| Common | 68 | 53 | 15 |
| LM vs AM | 4039 | 2032 | 2007 |
| LA vs AA | 2359 | 1288 | 1071 |
| Common | 1657 | 1005 | 652 |

Table S9. Quantitative Real-Time PCR (qRT-PCR) primers used in this study.

| Order | ID | Primer-F | Primer-R |
| --- | --- | --- | --- |
| 1 | c14462.graph_c0 | CGAGGGTACGAAAGTTCACACA | TGCTGTCGATTCGTCTGCTATT |
| 2 | c14905.graph_c0 | GGTTCGGAACATCCAGCAATAT | TCCTTCGGGAGTATCATCTTTGAG |
| 3 | c17020.graph_c0 | TCAAGCCTCCGTTGAAGCA | GAGTGCCCTCAAAAAACAAGGA |
| 4 | c17300.graph_c0 | ATCCGCAGAGCTCCTTACCA | GAGGCTAGACCGGAGAAAGAAAT |
| 5 | c18299.graph_c0 | GAGCAGCATATGAAGGTTGCATAC | TGGTCACTTGTGCTCTGAATGAAG |
| 6 | c19882.graph_c0 | GAAAATGACCGACCTGAAACCA | TCAAGCAACCCATTCTCTCCAT |
| 7 | c21711.graph_c0 | CAGACTATTGCAAGTCCACCATTG | AAACGACACAAAACAGCTCCAA |
| 8 | c27951.graph_c0 | TGCCGCCAGCAACTTTAATC | AACCATCGACTAGGGAGATATCGT |
| 9 | c28351.graph_c0 | AAGGAGGCATTGGTGTTGAGAT | CGCACAAGGAAGTCTCCATATG |
| 10 | c29417.graph_c0 | CAGATTGAAAGGCTCGGAGACT | CCGTCACTTCTTTCTACGCTAGTG |
| 11 | c34022.graph_c0 | GGCTATGCCTGGATTTTCAGAA | ATACTGCATTTCCCACCCTGTAG |
| 12 | c34108.graph_c0 | GAGACAAGGGAAGGCTGTCCAAT | TTGCTAGATGCCAAGGGTACCA |
| 13 | c35033.graph_c0 | TTTTGAGCGTCCCCAAGCT | GGAGATACCGAGAAAGACCAAGAA |
| 14 | c36087.graph_c0 | CAAGAGCTGTCCAAATGATCGA | GAGGGACAGAAAGCCCTATCAA |
| 15 | c36823.graph_c0 | TGTGCCGGAATTTCAAACGT | CATCGAAGATCTGTAAGCGCTATC |
| 16 | c36958.graph_c0 | TATGCACAGCCGATCTCAAAGC | TAAGATGCCCCCGTTTTCGT |
| 17 | c37205.graph_c0 | CGCCAGTCACATACAGGGAAT | GGGCCTCCTTTTTATCATCAGA |
| 18 | c39241.graph_c0 | GCTGAGACTGGAACCAGGAGAT | CCCTCCAAACCCACAGTGTTAA |
| 19 | c39469.graph_c0 | GCTTTCTGATGTTTCGGTTACACTT | TCCCCAACTATCATCATTCTTCCTA |
| 1 | c13055.graph_c0 | CCTTGCGCACAAGTGTAGCA | CAAGTTGTCGTACGAGTCGTTCA |
| 2 | c13379.graph_c0 | TTAGGACGGTCGATGGATTCA | CGCATTTTGGAAGCAACGAT |
| 3 | c13793.graph_c0 | AGCTGTATCCGATGGAGTCGAT | TGGGTGTTGGTGTTGACAATGT |
| 4 | c13833.graph_c0 | TGTCCGTACAACGTCTGGAATT | CAGGATCCGTCATTCCTACTCAT |
| 5 | c14326.graph_c0 | CTTCCTTCGTCTCCCAACATATG | CTCATCAATCCAGATGGCACTAGA |
| 6 | c14363.graph_c0 | TGTGCAATGATGACCCTAATGG | CATCTCTGTAGTCGAGGGTATCCA |
| 7 | c14491.graph_c0 | CACGCTTTGCATCAACCTACTG | CCAGCAACCGATACCATTCC |
| 8 | c14534.graph_c0 | CTCTAACTGGCTTCACGATGGA | TACCCTCCCCCTTCTTTCGTAT |
| 9 | c14787.graph_c0 | TCCTCCTCTCGTTCGATCAAA | AACGACTAACGCTGACCCAACT |
| 10 | c14854.graph_c0 | ATGGCATTCGTGGTGAGAAGT | CCAGTCTTTCACCCTAGGATCACT |
| 11 | c14905.graph_c0 | CCTTTCAGTGCAACCACGATAA | TGGTCCACTATTCGAACCTCTTC |
| 12 | c15308.graph_c0 | GCTGCAGCTGTGGGTTCATT | AAGGCAAGCCACCAAAGATG |
| 13 | c16233.graph_c0 | TGTGCAATGATGACCCTAATGG | CATCTCTGTAGTCGAGGGTATCCA |
| 14 | c16471.graph_c0 | GTGACAGAGCATCGTGGAATAACT | ACTGCATCGCTGATCAACATTG |
| 15 | c16660.graph_c0 | TCACGGACACCATGGTAGGA | TGGCGTTGTGGAGAATGTACTT |
| 16 | c16713.graph_c0 | AAGCGCGACAGGAATACTAGGA | CGTAACGAAGTTCGCAAATCTG |
| 17 | c17173.graph_c0 | TCCCCCTCATCCTCTGTATCTACT | CGATTGAGGAAAGGCACAATG |
| 18 | c17216.graph_c0 | AACTTGGGCTGCCATTTCG | GGCTAGGCACGTCAAAGAAGA |
| 19 | c17690.graph_c0 | ACGAAACCCGCCATCTTGT | GGTGCCGAGGGAAAAATATTC |
| 20 | c19860.graph_c0 | CGGCACAAAACCTCTCATCTC | TGGATAGCAATCCTCCACATGT |
| 21 | c21721.graph_c0 | TGCCAAAAGACCCCCAAA | AAGGTACCGGCATCGAAGAAC |
