# Supplementary figures and images for: Variation in life history traits and transcriptome associated with adaptation to diet shifts in the ladybird Cryptolaemus montrouzieri
Source: BMC Genomics. 2016 Apr 11;17:281. doi: 10.1186/s12864-016-2611-8 (PMC4827204; doi:10.1186/s12864-016-2611-8)

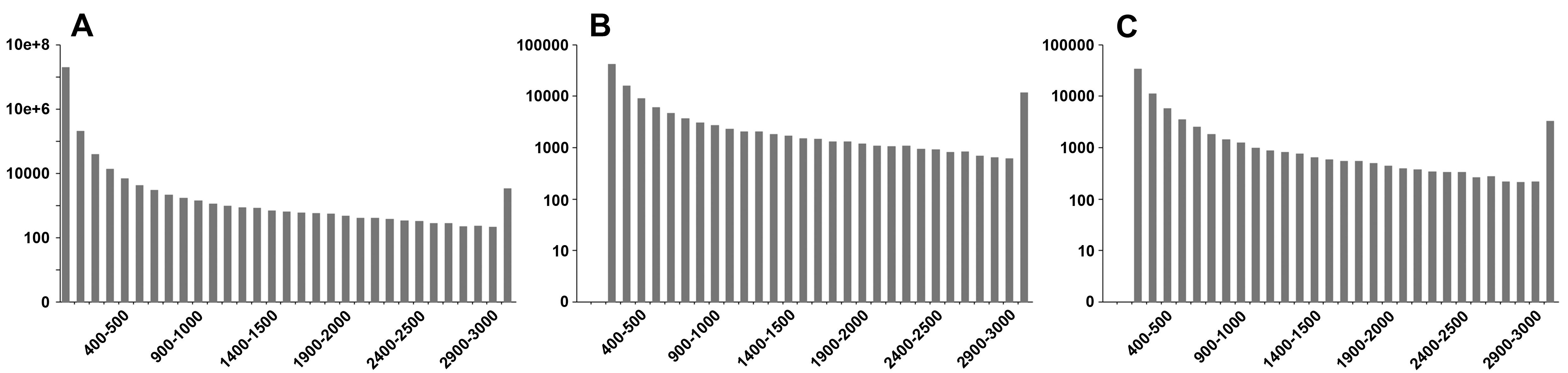

Supplement: Additional file 2: Figure S1. — Length distribution of the assembled A. contigs, B. transcripts and C. unigenes obtained from the combined reads of eight transcriptome libraries of C. montrouzieri. (TIF 774 kb) [file 12864_2016_2611_MOESM2_ESM.tif]

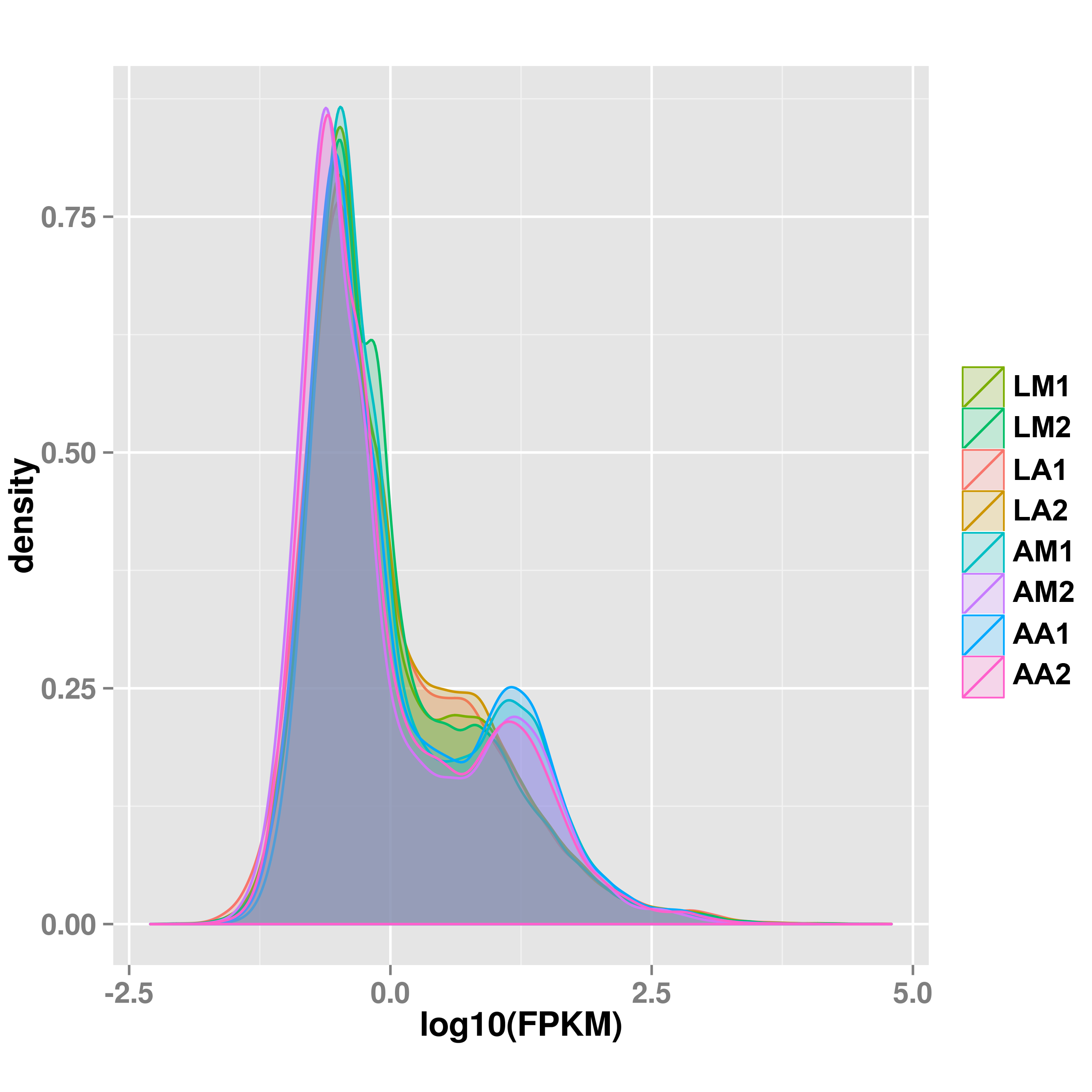

Supplement: Additional file 3: Figure S2. — Plot of fragments per kilobase of transcript per million mapped reads (FPKM) density of eight transcriptome libraries of C. montrouzieri. (TIF 804 kb) [file 12864_2016_2611_MOESM3_ESM.tif]

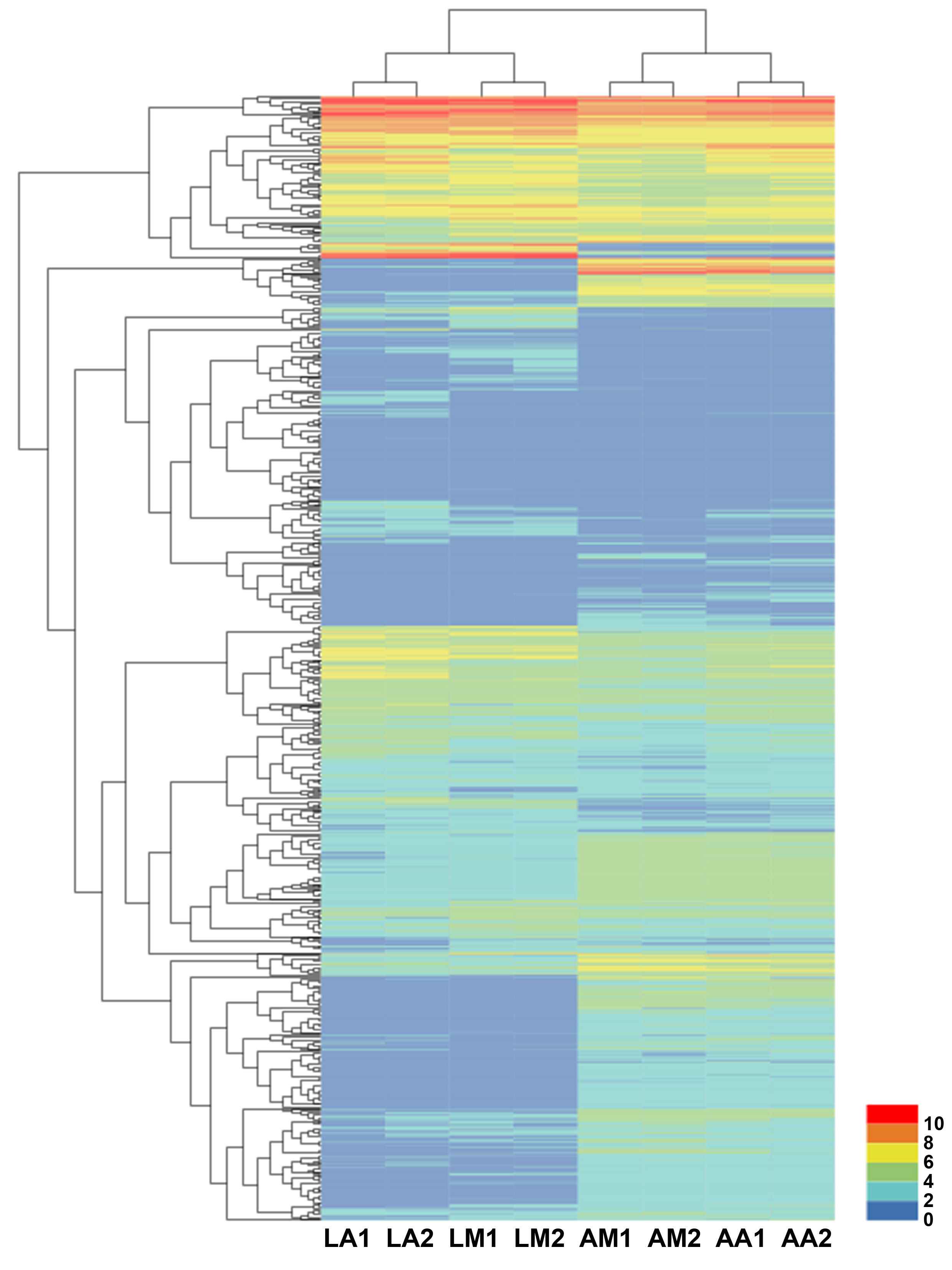

Supplement: Additional file 4: Figure S3. — Hierarchical clustering heat map of the gene abundance in eight transcriptome libraries of C. montrouzieri. Colours from red to blue represent the gene expression abundance from rich to poor. (TIF 1057 kb) [file 12864_2016_2611_MOESM4_ESM.tif]

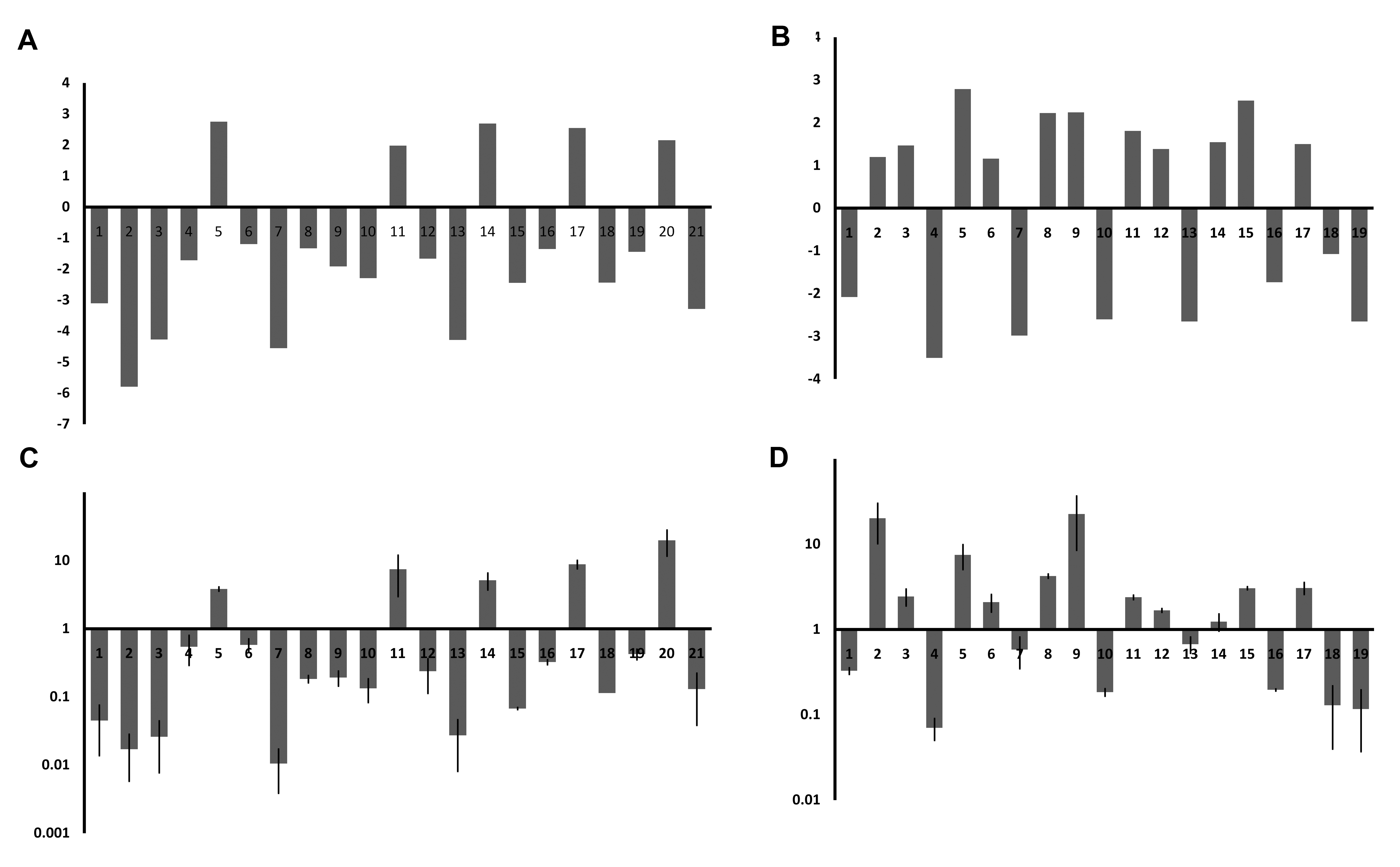

Supplement: Additional file 9: Figure S4. — Verification of differentially expressed genes (DEG) by quantitative Real-Time PCR (qRT-PCR). DEG data in transcriptome analysis of fourth instar larva (A) and adult female (B) and qRT-PCR analysis of fourth instar larva (C) and adult female (D) are compared. Fold differences in the expression of selected genes in response to diet shifts were calculated using the 2-ΔΔCt method. Data are presented as mean ± SD values of replicates for each gene transcript. Information of each gene number (1–21 of A and C and 1–19 of B and D) is shown in Additional file 1: Table S9. (TIF 656 kb) [file 12864_2016_2611_MOESM9_ESM.tif]
